# Voltage imaging in the olfactory bulb using transgenic mouse lines expressing the genetically encoded voltage indicator ArcLight

**DOI:** 10.1038/s41598-021-04482-3

**Published:** 2022-02-03

**Authors:** Jelena Platisa, Hongkui Zeng, Linda Madisen, Lawrence B. Cohen, Vincent A. Pieribone, Douglas A. Storace

**Affiliations:** 1 The John B. Pierce Laboratory, New Haven, CT USA; 2 Department of Cellular and Molecular Physiology, Yale University, New Haven, CT USA; 3 Department of Neuroscience, Yale University, New Haven, CT USA; 4 Allen Institute for Brain Science, Seattle, WA USA; 5 Brain Science Institute, Korea Institute of Science and Technology, Seoul, 136-791 Republic of Korea; 6 Department of Biological Science, Florida State University, Tallahassee, FL USA

**Keywords:** Olfactory bulb, Olfactory receptors, Multiphoton microscopy, Wide-field fluorescence microscopy, Imaging and sensing, Mouse, Fluorescent proteins, Optical imaging, Fluorescence imaging, Transgenic organisms

## Abstract

Genetically encoded voltage indicators (GEVIs) allow optical recordings of membrane potential changes in defined cell populations. Transgenic reporter animals that facilitate precise and repeatable targeting with high expression levels would further the use of GEVIs in the in vivo mammalian brain. However, the literature on developing and applying transgenic mouse lines as vehicles for GEVI expression is limited. Here we report the first in vivo experiments using a transgenic reporter mouse for the GEVI ArcLight, which utilizes a Cre/tTA dependent expression system (TIGRE 1.0). We developed two mouse lines with ArcLight expression restricted to either olfactory receptor neurons, or a subpopulation of interneurons located in the granule and glomerular layers in the olfactory bulb. The ArcLight expression in these lines was sufficient for in vivo imaging of odorant responses in single trials using epifluorescence and 2-photon imaging. The voltage responses were odor-specific and concentration-dependent, which supported earlier studies about perceptual transformations carried out by the bulb that used calcium sensors of neural activity. This study demonstrates that the ArcLight transgenic line is a flexible genetic tool that can be used to record the neuronal electrical activity of different cell types with a signal-to-noise ratio that is comparable to previous reports using viral transduction.

## Introduction

Genetically encoded voltage indicators (GEVIs) can report changes in membrane potential from defined cell types. The membrane localization, and the spatial and temporal characteristics of voltage signals make GEVI development challenging^1–6^. However, recent advances have improved GEVI sensitivity, speed, and signal-to-noise ratio^7–9^, allowing for enhanced detection of electrical transients in vivo^10–16^. While sensitivity is critically important, widespread adoption of GEVIs for in vivo experiments would benefit from the availability of tools that facilitate selective expression in specific cell types. To date, most mammalian in vivo approaches have driven expression using in utero electroporation^12,17^ or injection of a viral vector (e.g., adeno-associated viruses, AAVs)^10,11^. These protocols complicate the experiment by requiring additional surgical procedures, and in some instances, available viruses have limited transduction capacity for particular cell types (e.g., olfactory receptor neurons)^18^. Transgenic animals can overcome these limitations, as demonstrated by studies using GEVIs in *Drosophila* where bipartite systems such as GAL4/UAS allow for targeted expression in any brain region^19–23^. However, there are currently a limited number of transgenic mice expressing GEVIs with high signal-to-noise ratios (Butterfly 2.1^24^, QuasAr2^25^, ASAP2s^26^), and even fewer studies showing their utility *in vivo*^27,28^. Here we took advantage of a novel transgenic reporter mouse, Ai86(TITL-ArcLight)^24,26^, that can be used to selectively drive the expression of the GEVI ArcLight^7^ in specific cell types. The expression of ArcLight in the presence of both tetracycline transactivator (tTA) and Cre recombinase enhances cell-type specificity of this line^24,29^.

When ArcLight was first reported^7^, its fractional fluorescence change and signal-to-noise ratio were a substantial improvement over the then available GEVIs. Despite the development of several new GEVIs, a recent comparison reported that ArcLight remains the best GEVI for in vivo mammalian signals^17^. Similarly, Armbruster et al. (2021) reported that “subsequent experiments were performed using ArcLight exclusively due to its better (than Archon) signal-to-noise characteristics”^30^.

The olfactory bulb is the first region of information processing in the mouse olfactory system. The terminals of the olfactory receptor neuron input and the dendrites of the mitral/tufted output neurons both innervate olfactory bulb glomeruli. GEVIs have been used to study sensory processing in different olfactory bulb cell types using viral transduction as the expression vector^10,11^. Here we report a novel transgenic line with ArcLight expression localized to either olfactory receptor neurons (OMP-ArcLight) or olfactory bulb interneurons (Emx1-ArcLight) and demonstrate their ability to report odor-evoked signals in vivo.

## Material and methods

### Transgenic mice

All experiments were performed in accordance with the recommendations in the Guide for the Care and Use of Laboratory Animals of the National Institutes of Health, and in compliance with the ARRIVE guidelines. The protocol used was approved by the Institutional Animal Care and Use Committees of Yale University and The John B. Pierce Laboratory.

The ArcLight transgenic mouse line Ai86(TITL-ArcLight) (JAX Stock No. 034694) was generated by the Allen Institute for Brain Science^24,26^. A detailed description of the mouse line and the intersectional transgenic approach can be found elsewhere^24^. Ai86 is a Cre/tTA double reporter mouse line in which the GEVI ArcLight A242^7^ is knocked into the TIGRE locus (TIGRE 1.0). For this study, the Ai86(TITL-ArcLight) founder line was independently crossed to either Camk2a-tTA (Jax Stock #007004) or Emx1-Cre (Jax Stock # 005628) yielding double mutant lines Ai86 x CamK2a-tTA and Ai86 x Emx1-Cre, respectively. The transgenic offspring of these lines were crossed to create a triple transgenic mouse line Ai86 x Camk2a-tTA x Emx1-Cre (named Emx1-ArcLight) with ArcLight expression targeted to the granule cell layer and a small population of interneurons located in the glomerular layer in the olfactory bulb. Out of 178 pups produced in these crossings 28 expressed all three genes (15.7%). A second triple transgenic line with ArcLight targeted to the olfactory receptor neurons was created by crossing Ai86 x Camk2a-tTA and OMP-Cre (Jax Stock # 006668) lines, yielding Ai86 x Camk2a-tTA x OMP-Cre (named OMP-ArcLight). 11 out of the 45 offspring resulting from this cross were transgenic (24.4%). We confirmed the presence of ArcLight, tTA, and Cre using PCR-based genotyping performed either in-house or by Transnetyx (Cordova, USA) on all experimental animals. Adult mice were examined for apparent physical or behavioral abnormalities. Both lines were maintained for at least 2 years without any obvious changes in phenotype. Mice were housed under standard environmental conditions, with room temperature ranging between 23–25 °C and under a 12 h light/dark cycle. Our measurements were made during the light phase.

### Histology and confocal imaging

Following imaging, mice were euthanized (Euthasol) and brains (Emx1-ArcLight: N = 2; OMP-ArcLight: N = 1) were dissected and fixed in 4% paraformaldehyde for a minimum of 3 days. The OBs were embedded in 3% agarose and cut into 50–70 μm thick coronal slices on a vibratome. Sections were mounted on microscope slides using VECTASHIELD Mounting Medium with DAPI (Vector Labs, H-1500). Confocal images were obtained using a Zeiss LSM-780 confocal microscope (Carl Zeiss Microsystems, USA). The endogenous ArcLight fluorescence in histological sections was relatively bright and is presented without any amplification procedures.

### Surgical procedure

All surgical procedures were performed under sterile conditions. Male and female mice**,** ages between 30–180 days, were used for functional imaging experiments. Ketamine/xylazine (90 mg kg^−1^/10 mg kg^−1^) (Covetrus, USA) were used to induce and maintain a deep anesthetic state via intraperitoneal (IP) injections. Anesthetic depth was frequently assessed via the pedal reflex. A heating pad was used for the maintenance of body temperature at 37 °C throughout the procedure. Atropine (0.2 mg/kg, I.P., Covetrus, USA) was administered to reduce excessive bronchial secretion. The head was shaved and scrubbed with Betadine, and Lidocaine (0.5%) was injected into the skin above the dorsal olfactory bulbs. The skin above the cranium and olfactory bulbs was removed, and a custom head-post was fixed to the back of the skull using either cyanoacrylate or Metabond (Parkell, USA). The mouse was then mounted on a custom-made holder, which allowed for precise head positioning and fixation. Depending on the experiment, a high-speed dental drill (XL-230 Osada, Japan) was used to either thin (1-photon microscopy) or remove (2-photon imaging) the skull above both olfactory bulbs. For 2-photon imaging, the craniotomy was covered with 2% agarose and sealed with a #1 glass coverslip.

### Odorant stimuli and delivery

Different odorants (methyl valerate, isoamyl acetate, ethyl tiglate, and 2-heptanone; Sigma-Aldrich, USA) were used at concentrations between 0.12% and 11% of saturated vapor. A cleaned air stream was used for odorant dilution from saturated vapor. A flow dilution olfactometer ^31^ was designed to provide a constant airflow over the nares. A vacuum-controlled odorant delivery, where the vacuum was switched off during the odorant presentation. Separate Teflon tubing lines were used for each odor to avoid cross-contamination. In a subset of experiments, the time course and relative concentration of odors were confirmed with a photo-ionization detector (PID; Aurora Scientific, Aurora, ON). During imaging trials odorants were either delivered for 2–3 s with a 60-s delay between presentations, or odors were repeatedly presented with a 6-s interstimulus interval (adaptation trials).

### Imaging systems

The epifluorescence imaging was performed on a custom upright microscope equipped with a Prizmatix LED (UHP-T-LED-White-High-CRI), using either of two objectives a 35 mm F/1.4 Computar or a 25 mm F/0.95 Computar CCTV lens. We used a 479 nm (Semrock FF01-479/40) excitation filter combined with a 515 nm long pass dichroic mirror, and a bandpass filter. The neuronal activity was recorded with a NeuroCCD SM256 camera (RedShirtImaging, USA) with 2 × 2, or 3 × 3 binning and at a frame rate between 50–250 Hz. The images were collected with NeuroPlex software (RedShirtImaging, USA).

For 2-photon imaging, we used a modified MOM two-photon laser-scanning microscope (Sutter Instruments, USA) with a Nikon 16x, 0.8NA lens, a Coherent Discovery laser light source, and detected fluorescence emission on a GaAsP PMT (#H10770PA-40–04, Hamamatsu, Japan). 2-photon excitation of the super ecliptic pHluorin GFP chromophore was achieved with 940–980 nm laser light with an imaging speed of 31 frames per second (resonant scanners; Cambridge Technology, USA). The laser power was measured by placing a power meter (PM100D, Thorlabs) directly underneath the objective lens at the beginning of experiments and ranged between 75–140 mW.

### Data analysis

The data analysis was done in NeuroPlex, Excel and MATLAB. ArcLight reports voltage depolarization as decreases in fluorescence^7^, and all traces are inverted such that depolarizations are shown upwards. The presented optical traces are the spatial average of all the pixels within a region of interest (ROI). In the OMP-ArcLight transgenic mouse, individual glomeruli were visually identified in response to odor stimulation as glomerular-shaped peaks of activation ~ 50–100 μm in diameter using the Frame Subtraction feature of NeuroPlex. Response amplitudes for identified glomeruli were measured as the difference in the temporal average of the 1–2 s preceding the stimulus from a 0.8–1 s average around the peak of the response. Data are presented as the change in fluorescence divided by the resting fluorescence, ∆F/F. When necessary, the fluorescence traces were corrected to remove photobleaching by dividing the signal by a single exponential curve fitted to the portion of the trace prior to the stimulus. Pixels contaminated with blood vessel artifacts in major vessels were omitted from the analysis. The frame subtraction maps were generated in NeuroPlex using the Frame Subtraction function to subtract the temporal average of the 1–2 s preceding the stimulus from a 1 s temporal average around the response peak. The activity maps are displayed as depixelated (the depixelation function in NeuroPlex). The activity maps in Fig. 3 are average of 2–4 trials and are scaled to 90% of the maximum value at the highest odor concentration. In Fig. 6C, the activity maps are scaled to 97.5% of the maximum pixel value to emphasize the most active area, and the red contours were generated by carrying out a frame subtraction analysis in which the pixels with ∆F/F values between 95–100% of the maximum were identified. A contour was drawn around these pixels in Adobe Illustrator.

The error bars in Figs. 2, 3 and 5 and the statistics in the Results represent the standard error of the mean (s.e.m.). The baseline fluorescence analysis was carried out by measuring the mean fluorescence at the beginning of each trial. We restricted this analysis to a subset of our preparations in which we did not shift the imaging field of view and did not change the LED brightness. For the respiratory frequency analysis, the energy between 2–4.5 Hz was measured during the entire time period prior to the odor (~ 3 s), and during the odor presentation (~ 3 s) using the spectrogram function in MATLAB (Mathworks). The Hill coefficient analysis was carried out using the fit function in MATLAB where the half saturating odor concentration for each glomerulus was estimated by fitting the concentration versus response-amplitude points with an interpolated line, which was used to fit each glomerulus to the Hill equation:

$$\frac{{X}^{n}}{{X}^{n}+ {k}^{n}}$$

## Results

### Generating ArcLight transgenic mice

**Figure 1:**
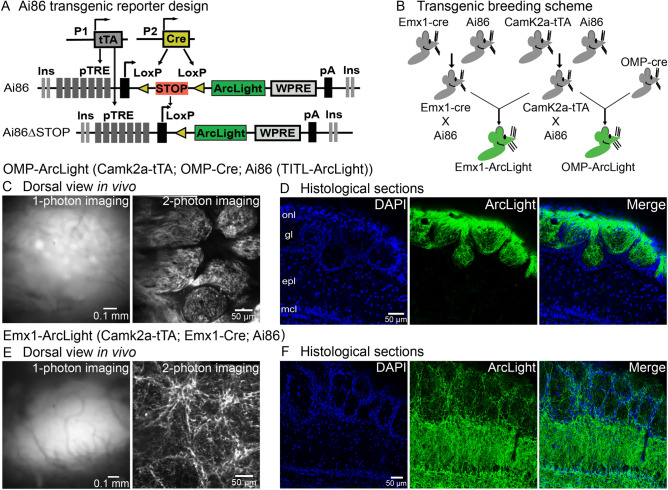
Expression of ArcLight in the olfactory bulb of transgenic mouse lines is robust and cell specific. (**A**) The design of the reporter line, Ai86 (TITL-ArcLight), is based on the Cre and tTA-dependent intersectional TIGRE1.0 approach. P1 and P2 are different promoters driving expression of Cre and tTA. (**B**) The breeding scheme used to create two triple transgenic mouse lines that show cell-specific expression of the GEVI ArcLight in the olfactory bulb. (**C, D**) OMP-ArcLight: (**C**) Dorsal view from the olfactory bulb in vivo using 1-photon imaging (*left panel*) and 2-photon imaging (*right panel*). (**D**) Histological sections illustrating that OMP-ArcLight transgenic mice has expression restricted to the olfactory nerve and glomerular layers. Similar 1-photon images were obtained in 6 different mice. (**E**, **F**) Emx1-ArcLight: (**E**) Dorsal view from the olfactory bulb in vivo using 1-photon (*left panel*) and 2-photon (*right panel)* imaging. (**F**) Histological sections illustrating that Emx1-ArcLight transgenic mice had ArcLight expression primarily located in the deeper granule cell layer and external plexiform layer, with some apparent labeling located in processes surrounding the glomeruli. Similar epifluorescence and 2-photon images were obtained in 5 different mice. onl, olfactory nerve layer; gl, glomerular layer; epl, external plexiform layer; mcl, mitral cell layer.

We generated two transgenic mouse lines in which the genetically encoded voltage indicator ArcLight was targeted to two different cell populations. These lines use the intersectional tTA/Cre TIGRE 1.0 system in which ArcLight is selectively expressed in cells that also express both tTA and Cre (Fig. 1A)^24,26^. A diagram of the breeding schemes used to generate the two lines is shown in Fig. 1B. The Ai86 (TITL-ArcLight) line was crossed to either the Camk2a-tTA or the Emx1-Cre mouse line, resulting in two new lines that either expressed ArcLight and tTA (ArcLight-tTA), or ArcLight and Emx1-Cre (ArcLight-Emx1) (Fig. 1B, *green*). The cross between these lines resulted in ArcLight expression in a population of olfactory bulb interneurons (named Emx1-ArcLight). A second line that expressed ArcLight in the olfactory receptor neurons (OMP-ArcLight) was generated by crossing the ArcLight-tTA and OMP-Cre lines. In vivo imaging from the mouse olfactory bulb was subsequently carried out in both transgenic lines.

### Robust and cell-specific expression of ArcLight in transgenic mouse lines

Individual glomeruli could be identified in OMP-ArcLight mice when imaging from the dorsal surface of the olfactory bulb using epifluorescence and 2-photon imaging from the glomerular layer in vivo (Fig. 1C). We confirmed in histological sections that ArcLight expression was restricted to the olfactory nerve and glomerular layer, and that no other cell types appeared to be labeled (Fig. 1D, N = 1 preparation). Resting fluorescence from the dorsal surface of the bulb in Emx1-ArcLight mice was diffuse under epifluorescence illumination, suggesting that the fluorescence was primarily localized outside of the glomerular layer (Fig. 1E). 2-photon imaging from the glomerular layer confirmed this observation as the glomeruli were weakly labeled, with higher expression levels present in neurons and processes surrounding each glomerulus (Fig. 1E, *right panel*). These observations were confirmed in histological sections in which the external plexiform layer was densely labeled with ArcLight fluorescence. This suggests a high expression of ArcLight in granule cells. Thus, the Emx1-ArcLight transgenic mouse has expression in interneurons located in the glomerular layer and the granule cell layer (Fig. 1F, N = 2 preparations). This result is consistent with a prior study indicating that Emx1 gives rise to a subpopulation of olfactory bulb interneurons^32^.

## OMP-ArcLight mice

### Ability of ArcLight to report respiratory modulation of receptor cell activity, and fluorescence stability

**Figure 2:**
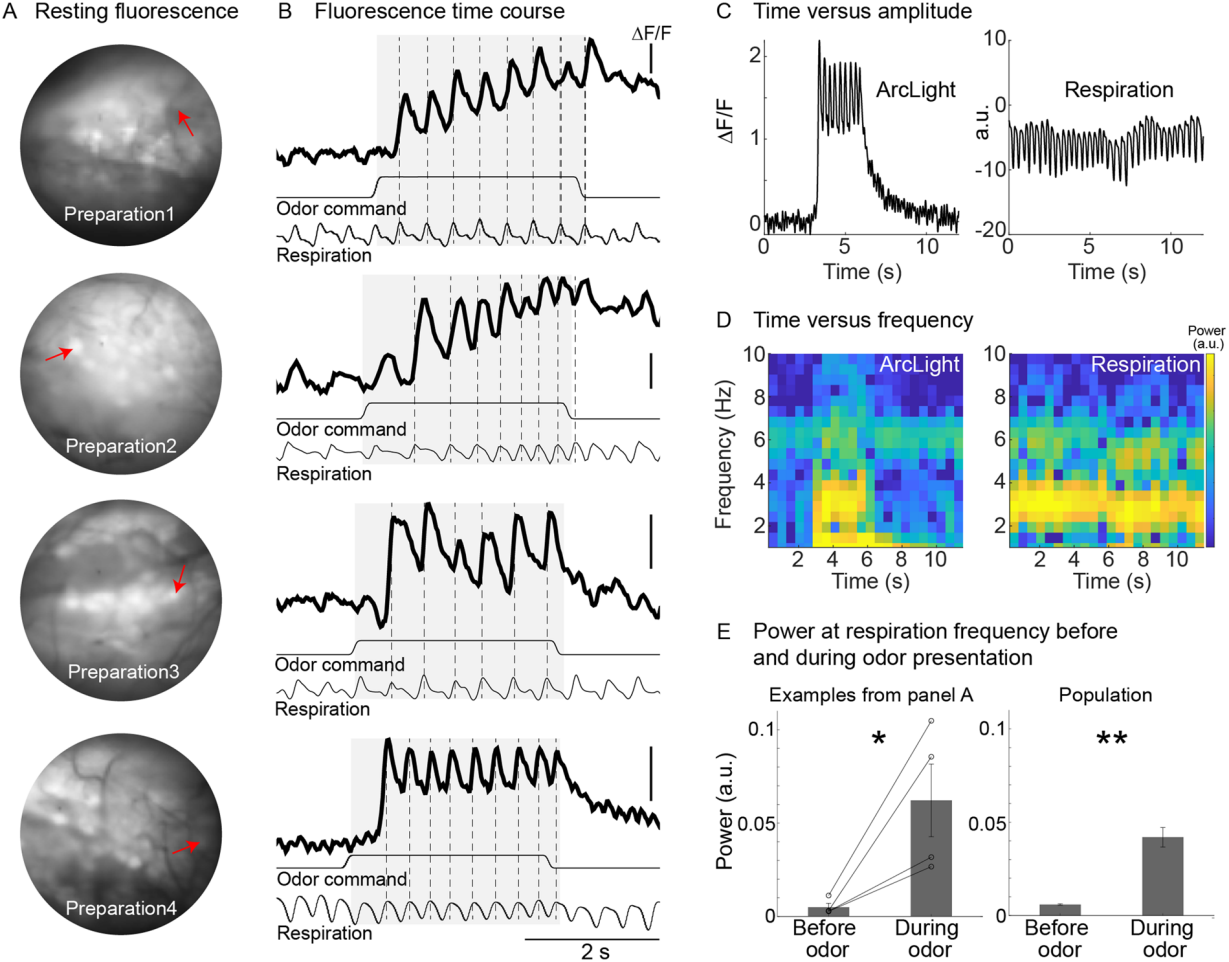
Odor-evoked activity in the olfactory receptor neurons (input) recorded with ArcLight in OMP-ArcLight transgenic mice. (**A**) Baseline fluorescence from four different preparations. (**B**) Single-trial recordings from the glomeruli indicated in panel **A** showing the ability of ArcLight to track the animal’s respiration. The traces are low pass filtered at 12 Hz. Each row is from a different preparation. Scale bars all indicate −1% ∆F/F. (**C**) Time versus amplitude plots for the ArcLight and respiration trace from the bottom preparation in panels **A**, **B**. (**D**) Time versus frequency graphs of the corresponding traces in panel **C**. (**E**) Average power at the respiratory frequency prior to and during odor presentation for the examples in **A**, **B** (*left panel*), and for a population of glomeruli (*right panel*). *p < 0.05; **p < 0.001.

Odors evoked relatively large changes in fluorescence that were tightly coupled to the animal’s respiration in subsets of glomeruli in anesthetized mice in vivo (Fig. 2B). To illustrate this relationship, we identified a subset of single trials in different preparations in which the animal’s respiration varied (Fig. 2B, *each row is a different preparation-glomerulus pair*, *dashed lines indicate respiration*). In all cases, the odor evoked large changes in fluorescence that were tightly coupled with the animal’s respiration. This is further evident by comparing plots of time versus amplitude (Fig. 2C) and time versus frequency (Fig. 2D) of the ArcLight signal and the animal’s respiration. The respiration trace had relatively high power between 2–4 Hz (Fig. 2C,D, *right panels*), a typical respiratory rate for anesthetized mice. The ArcLight signal exhibited a large increase in power in that frequency range during the odor presentation (Fig. 2C,D, *left panels*). Overall, there was a significant increase in power at the respiratory frequency for the individual glomeruli in Fig. 2A,B (Fig. 2E, *left panel,* *p* < 0.05, ranksum = 10, N = 4 glomeruli), and across a population of glomeruli (Fig. 2E, *right panel*; *p* < 0.001, ranksum = 28, 43 glomeruli in 5 preparations). The respiratory modulation detected with ArcLight was substantially larger than that seen with GCaMP3 and GCaMP6f^10^.

The ArcLight baseline fluorescence and odor-evoked activity did not diminish during imaging sessions of up to 2 h. The average baseline fluorescence did not significantly change, which was quantified by measuring the baseline fluorescence across the entire bulb during an imaging trial occurring at the beginning of the experiment, and one at the end of the experiment (Initial: 4621 ± 293 a.u., Final: 4514 ± 384, p = 0.82, N = 7 preparations). These results demonstrate that ArcLight reports odor-evoked activity in the mouse olfactory bulb in vivo with a high signal-to-noise ratio and sufficiently fast temporal kinetics that reports respiratory coupled activity. Moreover, the baseline fluorescence of ArcLight does not undergo significant bleaching and can thus be used to carry out in vivo imaging across relatively long imaging sessions. These results are consistent with our prior work in which ArcLight was expressed using adeno-associated viral transduction^10^.

### Concentration-dependence of odor-evoked activity in olfactory receptor neuron glomeruli

**Figure 3:**
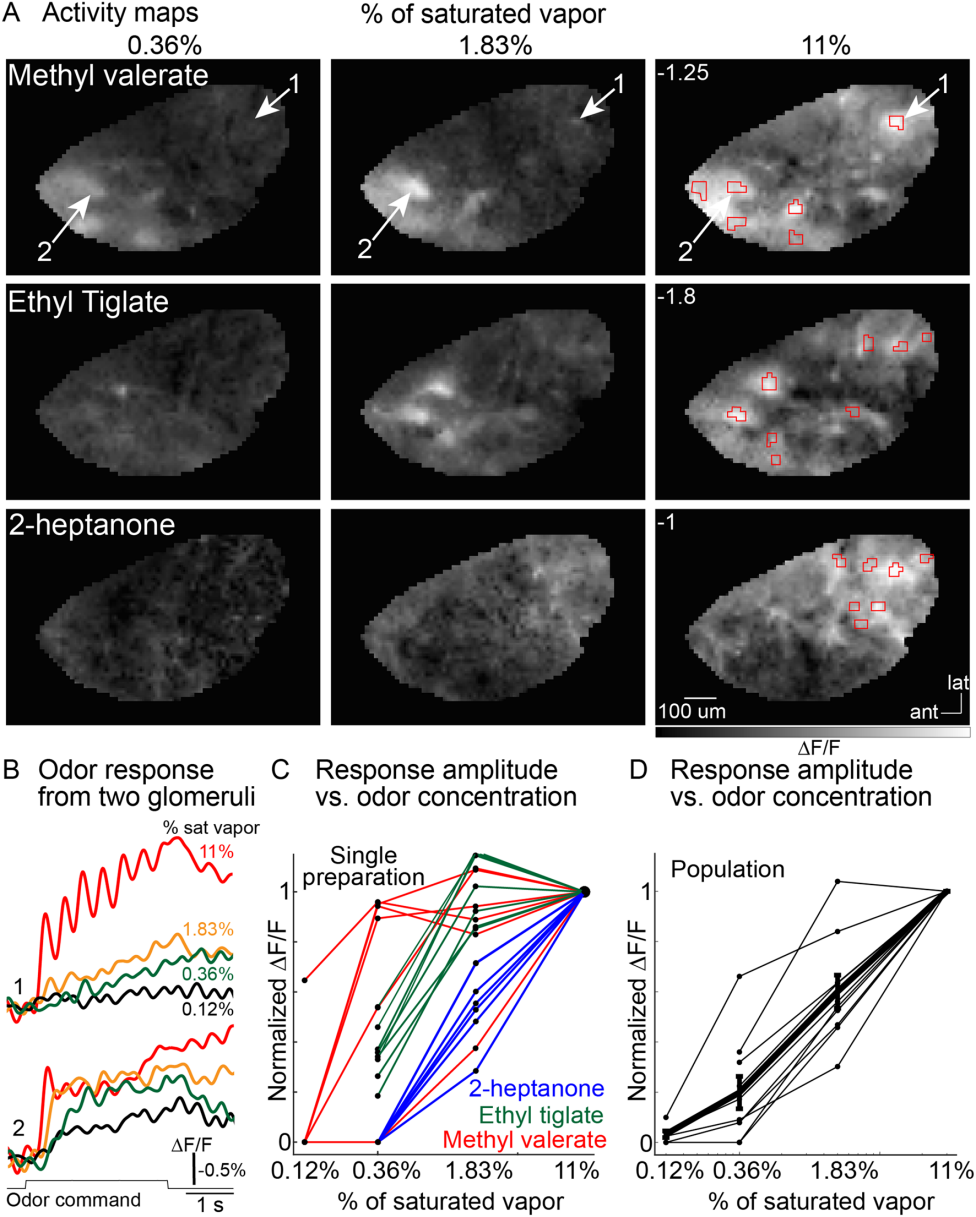
Responses of OMP-ArcLight glomeruli to different odor-concentration pairings. (**A**) Activity maps evoked by different concentrations of methyl valerate, ethyl tiglate and 2-heptanone. The maps are scaled to 90% of the maximum pixel value evoked at the highest concentration of each odor (max value indicated as white text on the 11% of saturated vapor panels). (**B**) Fluorescence time course from glomeruli 1 and 2 in response to methyl valerate at 0.12, 0.36, 1.83 and 11% of saturated vapor. The regions of interest are indicated by the arrows in panel **A**. (**C**) Normalized signal amplitude versus odorant concentration for all the glomeruli recorded in panels **A**, **B**. (**D**) Normalized signal amplitude vs odorant concentration for 7 preparations where the responses from all the activated glomeruli were averaged together for each preparation.

*Different odor-concentration pairs*. Responses to different odor-concentration pairs were imaged across the dorsal OB using epifluorescence imaging. A frame subtraction analysis was used to generate activity maps that demonstrate that different odors evoked distinct glomerular-sized peaks of activity. The results from a single preparation in which the response to three odors were measured across the same concentration range are illustrated in Fig. 3A. The fluorescence time course from two glomeruli in response to the odor methyl valerate presented between 0.12 and 11% of saturated vapor are shown in Fig. 3B (*glomeruli indicated by arrows in the top row*). Increasing the odor concentration caused increases in both the number of activated glomeruli (Fig. 3A) and the response amplitude (Fig. 3B).

The response amplitude evoked by each glomerulus-odor pair was measured (e.g., Fig. 3B) and normalized to the response evoked by the highest odor concentration (11% of saturated vapor). The concentration–response amplitude for each glomerulus-odor pair from the preparation in Fig. 3A,B is plotted against concentration in Fig. 3C (colors indicate odor). The response amplitude for all odor-glomerulus pairs within a preparation were averaged together (Fig. 3D, *thin lines,* each line reflects the measurement of 3–11 glomeruli, 10 preparation-odor pairs across 6 preparations). The thick black line in Fig. 3D illustrates the mean response across all preparation-odor pairs. Each of the preparation-odor pairs were fit to the Hill equation, and the Hill coefficient ranged between 1.03 and 2.2 (mean ± s.e.m. was 1.65 ± 0.12).

Overall, ArcLight measurements from olfactory receptor neuron glomeruli demonstrate that different odors evoked distinct activity maps and that they exhibited a steep concentration–response relationship. Thus, olfactory receptor neuron glomerular measurements carried out using the voltage sensor ArcLight were similar to previous reports using organic calcium dyes, protein sensors of calcium, and synaptic vesicle release^11,33,34^.

### Temporal diversity of odor-evoked signals across olfactory bulb glomeruli

**Figure 4:**
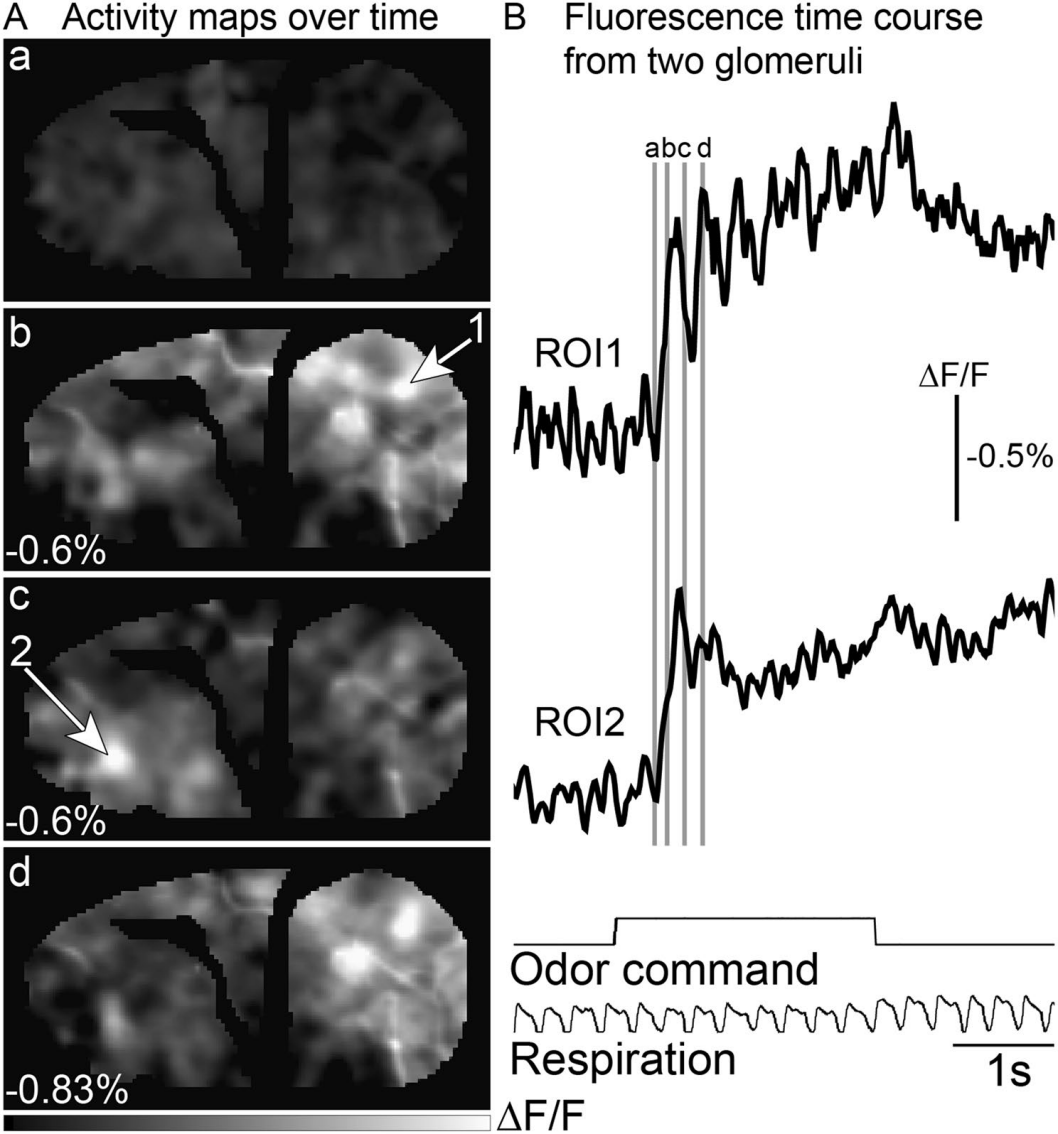
Dynamics of odor-evoked signals. (**A**) Activity maps were measured at 4 different time points: prior to odor (*a*), and at three time points (*b*-*d*) following the animal’s inspiration of the odor stimulus. The maps are scaled to 90% of the maximum value pixel value (number in the bottom left corner of each panel). (**B**) Fluorescence time course of odor-evoked signals taken from glomeruli located in the caudal and rostral bulb, respectively (ROIs indicated by the arrows in panel **A**). The black trace is the average of two single trials aligned to the first breath. The traces are low pass filtered at 12 Hz. The time points used for the activity maps in panel **A** are indicated by the gray lines.

Olfactory receptor neuron glomeruli located in the caudal and rostral parts of the olfactory bulb show distinct temporal dynamics^35^. We carried out a frame subtraction analysis at four time points relative to the odor presentation, which demonstrates that the glomerular activation pattern varies dynamically across time (Fig. 4A). Fluorescence traces from two different glomeruli are illustrated in Fig. 4B (the gray bars indicate the time points used to generate the activity maps in Fig. 4A). Caudal glomeruli tended to be tightly coupled to respiration, returning toward the baseline between inhalations (Fig. 4B, *ROI1*). In contrast, those located more anteriorly were slower to reach their peak response and had less respiratory modulation (Fig. 4B, *ROI2*). These results demonstrating distinct temporal dynamics between glomeruli in different parts of the bulb are consistent with prior reports using voltage and calcium sensors^10,35,36^.

### Adaptation in the olfactory bulb input

**Figure 5:**
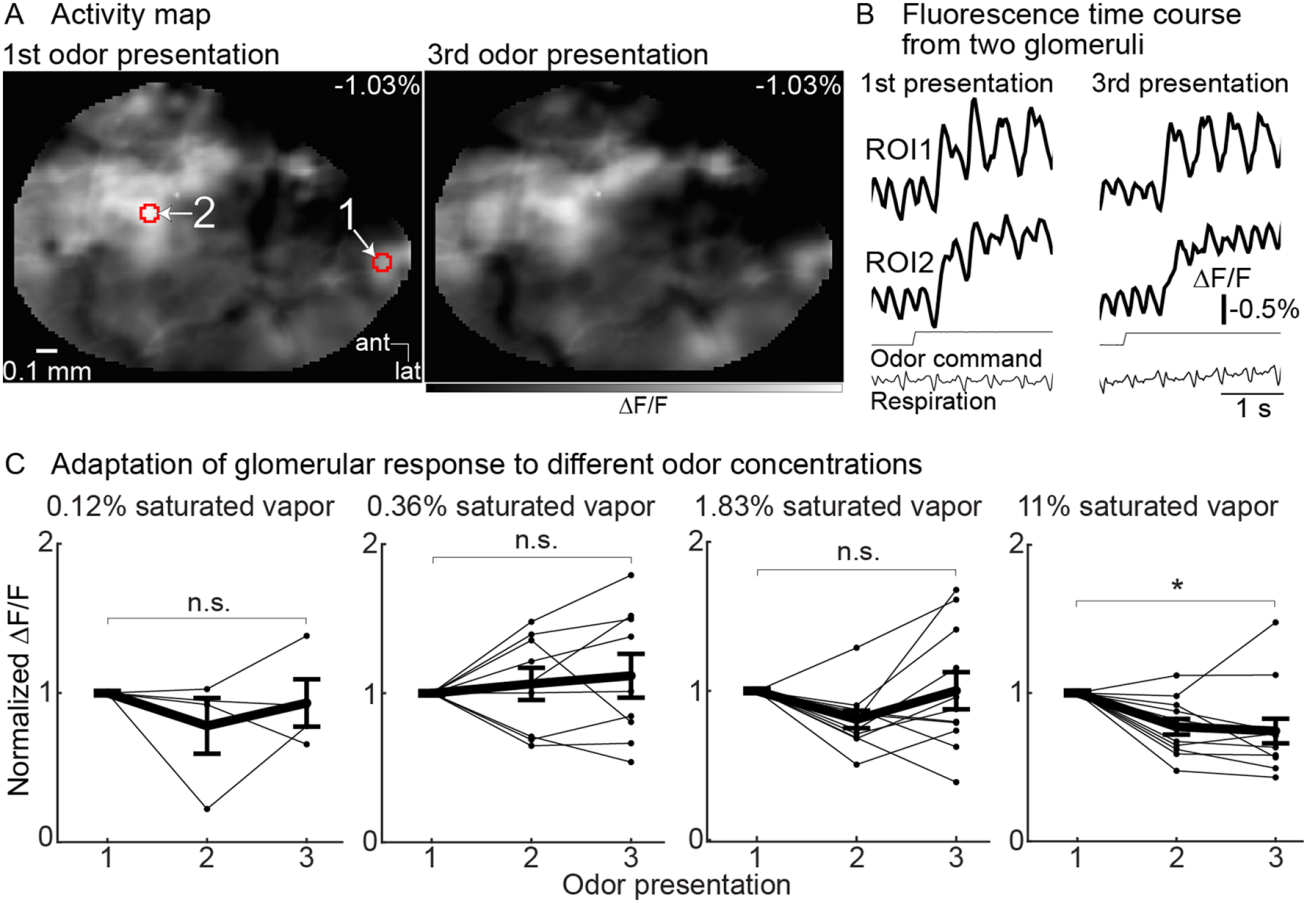
Olfactory receptor neuron glomeruli exhibit minimal adaptation in response to repeated 3-s presentations separated by a 6-s interval. (**A**) Activity map of neuronal activity evoked by 1.83% of saturated vapor (methyl valerate) in response to the 1st (*left panel*) and 3rd (*right panel*) odor presentation. The maps are scaled to the same min and max range. (**B**) Fluorescence traces from the two glomeruli indicated in panel **A**. The traces are cropped to illustrate the response to the 1st (*left column*) and 3rd (*right column*) odor presentation and are low pass filtered at 12 Hz. (**C**) Population normalized response amplitude of repeated odor presentations at four different odor concentrations. Each line indicates the mean response of all activated glomeruli to an odor from a single preparation. The mean ± s.e.m. responses across all preparations are shown as thick black lines. *p < 0.01.

Prior studies have reported that olfactory receptor neurons exhibit modest adaptation over time periods of 5–10 seconds^37–41^. We examined the adaptation of olfactory receptor neurons by measuring the response to repeated 3-s odor presentations separated by a 6-s interstimulus interval. Figure 5A illustrates an activity map evoked by the odor methyl valerate (1.83% of saturated vapor) in response to the 1st and 3rd odor presentation. The overall pattern and amplitude are relatively similar to one another (the maps are scaled to the same range). Single-trial fluorescence time course from the two glomeruli identified by the red polygons in panel **A** are shown in Fig. 5B. Adaptation was measured in 188 odor-glomerulus pairs (7 preparations, 3–11 glomeruli per preparation, 1–3 odors tested per glomerulus, 3–4 concentrations tested per odor).

All odor-glomerulus pairs were averaged together from each preparation and examined as a function of odor concentration (Fig. 5C). The response to each odor presentation within a trial was normalized to the amplitude evoked by the 1st stimulus. The mean population response showed a significant reduction to the 3rd pulse at the highest odor concentration (Fig. 5C, 11% of saturated vapor, 3rd pulse: 0.74 ± 0.08, *p* < 0.01, 12 preparation-odor pairs in 7 preparations). However, glomerular signals evoked by lower concentrations were relatively stable across repeated odor presentation (1.83% of saturated vapor, 3rd pulse: 1 ± 0.12, p = 0.26; 11 preparation-odor pairs in 6 preparations; 0.36% of saturated vapor, 3rd pulse: 1.1 ± 0.14; p = 0.7, 9 preparation-odor pairs in 5 preparations; 0.12% of saturated vapor, 3rd pulse: 0.93 ± 0.15; p = 0.31, 4 preparation-odor pairs in 4 preparations; all values are normalized ∆F/F).

These results are consistent with prior reports using calcium sensitive sensors indicating that olfactory receptor neurons exhibit adaptation at very high odor concentrations but recover in response to odor stimuli at low-to-moderate concentrations with interstimulus intervals of 6 seconds^39,40,42,43^.

### Emx1-ArcLight mice

**Figure 6:**
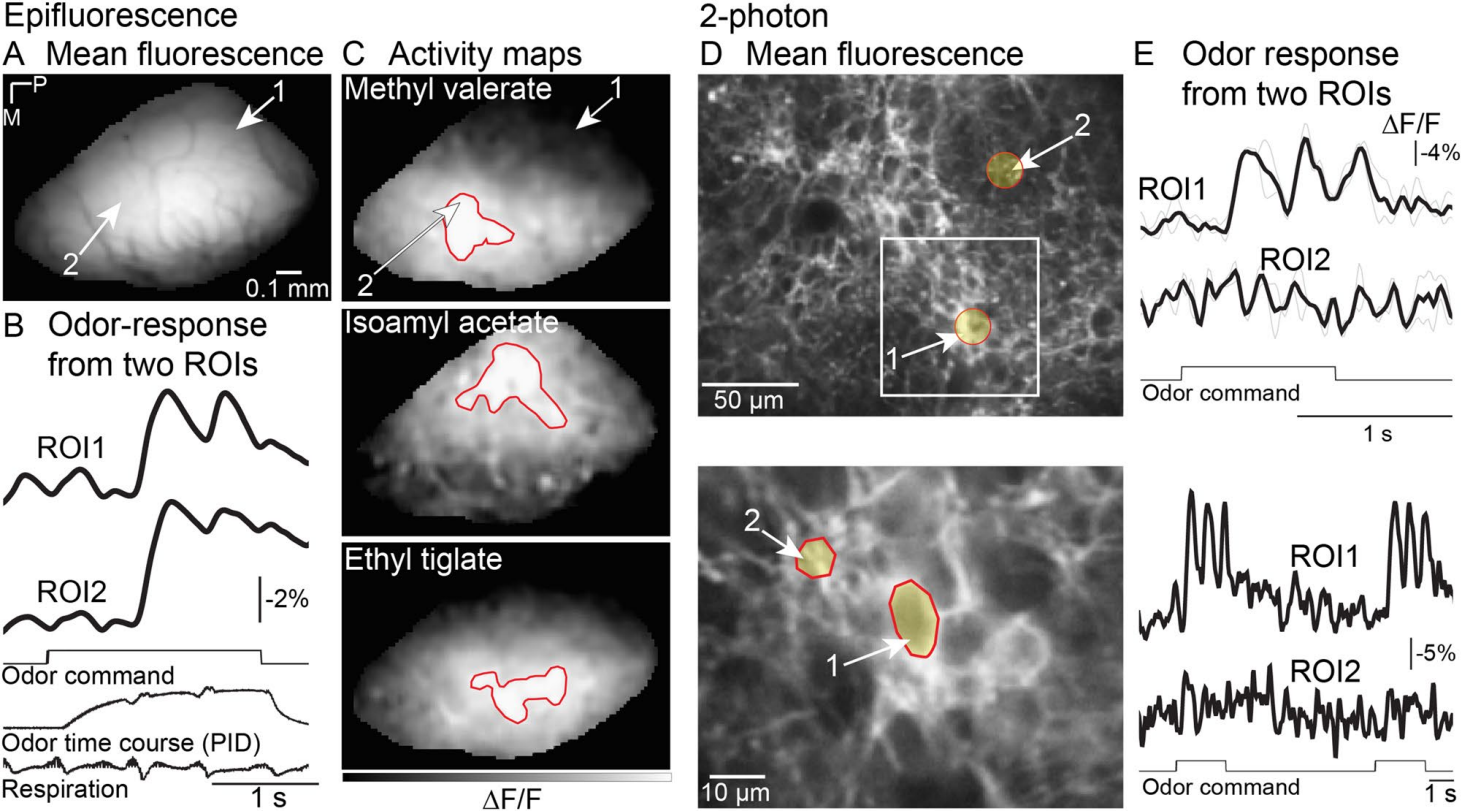
In vivo imaging from the Emx1-ArcLight transgenic mouse. (**A**-**C**) Epifluorescence imaging. (**A**) Resting fluorescence from the olfactory bulb. (**B**) Fluorescence traces from two regions of interest (ROIs indicated by arrows in panel **A**). Traces are low pass filtered at 4 Hz. (**C**) Activity maps evoked by three different odors. Activity maps are spatially filtered with one 3 × 3 iteration of a gaussian low-pass filter. The red contours indicate the area with ∆F/F values between 95–100% of the maximum response. (**D**, **E**) 2-photon imaging. (**D**) (*top*) Resting fluorescence from the glomerular layer illustrating weak expression in the glomeruli, and brighter fluorescence on the edges of the glomeruli. (*bottom*) A higher magnification recording from the area illustrated by the white box. (**E**) (*top*) Odor-evoked activity could be detected from the areas of bright expression around the glomeruli. (*bottom*). The signals are low pass filtered at 2 Hz. The black traces in the top panel are an average of two trials (gray lines). The black traces in the bottom panel are single trials. P, posterior; M, medial.

*Wide-field imaging.* Similar imaging experiments using epifluorescence microscopy were carried out using the Emx1-ArcLight transgenic line. Unlike the OMP-ArcLight mouse, the baseline fluorescence in Emx1-ArcLight mice was diffuse and individual glomeruli could not be observed (Fig. 1E, 6A). Odors evoked relatively large and diffuse (i.e., non-glomerular) changes in fluorescence across the dorsal bulb (Fig. 6C). The lack of glomerular peaks of activity was also consistent with the histology showing minimal fluorescence located in glomeruli (Fig. 1F). Despite the diffuse signal, different odors could broadly evoke activity in different parts of the dorsal bulb (Fig. 6C, *compare the contours drawn for each odor*). In addition, some differences in temporal dynamics could be distinguished when comparing regions of interest taken from the caudal and rostral bulb (Fig. 6B, *ROI1 vs ROI2*). This observation is consistent with previous studies indicating that some of the temporal differences evident in olfactory receptor neuron glomeruli are also present postsynaptically (Fig. 4)^35,36,44^.

*Two-photon imaging.* Although histological examination of fixed sections confirmed that individual cells were labeled in the glomerular layer in Emx1-ArcLight mice (Fig. 1F), they could not be resolved using epifluorescence imaging due to light scattering and diffuse fluorescence originating from the deeper layers. Previous studies have shown that ArcLight is compatible with 2-photon imaging, which dramatically reduces the contribution from signals above and below the focal plane^10,17,45^. Here we asked whether it was possible to resolve activity from these neurons in vivo using 2-photon microscopy.

The average fluorescence imaged from the glomerular layer using 2-photon imaging is illustrated in Fig. 6D. Although glomeruli had relatively weak fluorescence (Fig. 6D, *top, #2*), the regions surrounding the glomeruli were brightly labeled and in many cases were consistent with individually labeled neurons with localized membrane fluorescence (Fig. 6D, *top #1*). Odor-evoked responses were not easily detectable from glomerular regions of interest (Fig. 6E, *top*), although they were detected in single trials in the regions surrounding the glomeruli (Fig. 6D, *arrow #1*).

We imaged at a higher magnification to examine the glomerular surrounds more closely, which revealed regions of interest that had the same size as a cell (Fig. 6D, *bottom*). Odors evoked clear responses from the cell-like region of interest while neighboring regions exhibited no detectable signal (Fig. 6E, *bottom*, compare ROIs 1 and 2). Qualitatively similar results were observed in 5 additional preparations. Thus, ArcLight in this transgenic mouse can be used to detect odor-evoked signals that are apparently from individual neurons in the olfactory bulb using 2-photon imaging.

## Discussion

The GEVI ArcLight is a chimeric voltage indicator^7^ made of a voltage-sensitive domain derived from the *Ciona intestinalis* voltage-sensitive phosphatase^46,47^ and a mutated version (A227D)^7^ of super ecliptic pHluorin, a GFP^48,49^. Neuronal cells that express ArcLight exhibit a depolarization-dependent decrease in fluorescence intensity. While ArcLight has relatively slow kinetics (~ 10 ms fast time constant) which limits its ability to detect high-frequency spiking activity^50^, it is bright, sensitive, and has been shown to respond to neuronal activity in vivo in *C. elegans*, *Drosophila*, mouse, and even plants^10,11,17,19,22,23,51–53^. Previous imaging studies using ArcLight in mice used either in utero electroporation^12,17^ or AAV viral vectors under the control of different promoters (hSyn and CAG)^10,11^.

The ArcLight transgenic mouse line, Ai86(TITL-ArcLight), was developed as a part of an effort to increase the availability of transgenic mice that can be used to drive high levels of specific cell-type expression of various fluorescent reporters^24,26^. Here, we used wide-field epifluorescence and 2-photon microscopy to functionally characterize two de novo generated mouse lines with distinct patterns of ArcLight expression in the olfactory bulb (OMP- and Emx1-ArcLight). These mouse lines allowed us to examine different aspects of olfactory bulb physiology, including odor-evoked activity maps, temporal dynamics, concentration dependence, and adaptation.

While voltage imaging has been previously carried out from olfactory receptor neuron glomeruli in vertebrates^54^, this study provides the first report of its use in the mammalian olfactory bulb. Our prior attempts to use nasal infusion of voltage dyes to record from olfactory receptor neurons resulted in bright labeling, but we were unable to detect odor-evoked signals in vivo (di-8-ANEPPS; unpublished observation, D.A.S. and L.B.C.). Furthermore, attempts to use several AAVs expressing various indicators to transduce olfactory receptor neurons via nasal infusion resulted in little to no expression in our hands (unpublished observation, D.A.S. and L.B.C.) and others^18^. Thus, the OMP-ArcLight transgenic line represents the first in vivo measurements of membrane potential changes from olfactory receptor neuron glomeruli in mice*.* Because previous measurements of the odorant concentration dependence and adaptation in olfactory receptor neurons had been carried out with calcium sensitive indicators^11,33,34,42^, there was concern that those results might depend on the use of calcium as a surrogate for action potential activity. Using the OMP-ArcLight mice, we found that the voltage indicator results (Figs. 2–5) were essentially identical to those reported using calcium indicators.

The 2-photon imaging experiments were consistent with our prior report in which ArcLight could reliably report odor-evoked activity^10^. Here we found several examples that exhibited a cell-like appearance in the resting fluorescence in the regions surrounding olfactory bulb glomeruli in the Emx1-ArcLight transgenic line (e.g., Fig. 1F). A prior study crossed the Emx1-cre transgenic line to an eGFP reporter line and found labeled cells in the granule and glomerular layers. This result is consistent with the expression in our Emx1-ArcLight transgenic line^32^. Moreover, Kohwi et al. (2007) demonstrated that the glomerular layer interneurons included a subset of periglomerular cells, consistent with their anatomical location within the olfactory bulb. Regardless, we are uncertain whether the cell-like regions of interest found in our Emx1-ArcLight transgenic mouse are the same as described in this prior report, and thus we interpret the results cautiously. Future experiments are needed to confirm the genetic identity of this interneuron subpopulation. Moreover, because the ArcLight sensor was expressed throughout the periglomerular regions, it was difficult to confirm that the signal was indeed restricted to a single cell body. In the future, the application of targeting sequences that restrict GEVI expression to the neuronal membrane of the cell body will make it easier to confirm single-cell responses^16,55,56^.

Our experiments were carried out in mice that were anesthetized with ketamine/xylazine, an antagonist for NMDA receptors^57^ which play an important role in olfactory bulb signaling^58–60^. Anesthesia is known to alter the physiology of mitral and tufted cells^36,61^, thus it is possible that the anesthesia used in these experiments may have impacted our measurements. However, in preliminary experiments, we imaged odor responses from the OMP-ArcLight transgenic line while anesthetized, and again during wakefulness and found that the activity maps were qualitatively similar (J.P., L.B.C. and D.A.S., *preliminary results*). In another comparison, the olfactory bulbs of anesthetized and awake mice had similar adaptation properties^42^. Overall, additional awake-anesthetized comparisons may prove informative.

The original discovery of ArcLight led to a remarkably productive decade in which several laboratories have developed a catalog of GEVIs with varying biophysical (e.g., fluorescent protein vs opsin) and optical properties (i.e., sensitivity, signal-to-noise ratio, brightness, color)^1,4^. Although the specific choice of GEVI will depend on the application and preparation type^1^, ArcLight provides a bright and stable GEVI with a high signal-to-noise ratio that has been shown to work in multiple model organisms^10,19,51,52^, and in two different sensory systems in the mouse^11,53^. Indeed, in a comparison of GEVIs, ArcLight was found to have the largest signal-to-noise ratio^17^, and unlike opsin-based probes that do not function under 2-photon excitation^17^, ArcLight works when using both epifluorescence and 2-photon imaging (Fig. 6)^10,21^. Moreover, the ArcLight transgenic line is one of the few options for using transgenic mice in vivo.
